# T Cell Subsets in HIV Infected Patients after Successful Combination Antiretroviral Therapy: Impact on Survival after 12 Years

**DOI:** 10.1371/journal.pone.0039356

**Published:** 2012-07-17

**Authors:** Frederikke Falkencrone Rönsholt, Sisse Rye Ostrowski, Terese Lea Katzenstein, Henrik Ullum, Jan Gerstoft

**Affiliations:** 1 Department of Infectious Diseases, Rigshospitalet, Copenhagen, Denmark; 2 Department of Clinical Immunology, Rigshospitalet, Copenhagen, Denmark; Salute San Raffaele University School of Medicine, Italy

## Abstract

**Objectives:**

Immune activation is decreased by combination antiretroviral therapy (cART) in patients infected with human immunodeficiency virus (HIV), but residual activation remains and has been proposed as a cause of premature aging and death, but data are lacking. We analyzed the relationship between T-cell subsets after 18 months of cART and overall survival during 12 years of follow up.

**Methods:**

A cohort of 101 HIV infected patients who had undetectable plasma HIV after starting cART was included in 1997–1998. T cell subsets were analyzed by flowcytometry after 18 months of cART. Relation to survival was calculated using Kaplan-Meier curves and multiple Cox regression.

**Results:**

Seventeen patients died during the observation period. The leading causes of death were non-AIDS cancer and cardiovascular disease. Higher levels of CD8 memory T cells (CD8+,CD45RO+,CD45RA-) showed a significant beneficiary effect on survival, HR of 0.95 (95% confidence interval 0.91–0.99, P = 0.016) when adjusted for age, nadir CD4 count, CD4 count, and AIDS and hepatitis C status. T cell activation was not associated with increased risk of death.

**Conclusions:**

Larger and longitudinal studies are needed to accurately establish prognostic factors, but overall results seem to suggest that prognostic information exists within the CD8 compartment.

## Introduction

Since the discovery and implementation of combination antiretroviral therapy (cART) the prognosis of human immunodeficiency virus (HIV) infected patients has improved enormously. As the HIV-infected patients’ life-expectancy increases and the risk of developing AIDS decreases the focus of attention is moving toward a documented excess non-AIDS morbidity and mortality due to cardiovascular disease and certain types of cancer. Several, probably coexistent, factors have been suggested as contributors to this, including excess of risk factors such as smoking, cART toxicity and foremost the theory that ongoing immune activation/dysfunction leads to chronic inflammation that causes comorbidity and mimics natural aging at the cellular level (“inflamm-aging” or “immunosenescence”) [1], [2].

Studies in patients nor receiving cART have convincingly shown that increased levels of cellular and soluble markers of immune activation are linked to higher risk of progression to AIDS, lower CD4 counts and poorer prognosis [3]–[6]. Among the cellular markers particularly measurements of CD8+CD38+ T cells have demonstrated prognostic value in untreated patients [7], [8]. cART effectively induces viral suppression and a partial restoration of immune function and composition, but residual immune activation is the rule [9], [10]. This residual immune activation has also been shown to impair immune reconstitution [11]–[14]. However, the clinical importance of this residual immune activation, microbial translocation and T cell senescence remains undetermined as most of the studies addressing this crucial question are either cross-sectional, longitudinal with limited follow-up time or relies on surrogate laboratory endpoints.

We aimed to address the relationship between T cell subsets measured in 1997-98 18 months after start of successful cART and the overall survival during 12 years of follow-up in a cohort of HIV infected patients.

## Materials and Methods

### Population

The study was conducted at the Department of Infectious Diseases and the Department of Clinical Immunology at Rigshospitalet (Copenhagen, Denmark). The study population consisted of HIV infected patients included in the period September 1997–August 1998 on the basis of having reproducible plasma HIV RNA levels <200 copies/mL after starting cART. One-hundred-and-one patients with an immunological investigation done 18 months after cART initiation were included in this study. All patients gave written informed consent and the study was approved by the Ethics Committee.

As treatment interruptions have never been part of the Danish treatment guidelines, the patients in this cohort have been offered cART continuously since the inclusion in 1997, although the drug combinations may have changed over the years due to drug development, side effects etc.

This group of well treated patients underwent repetitive extensive immunological examinations at regular intervals for the first 24 months of treatment [15]–[19]. For the purpose of this study baseline was defined as the date of immunological evaluation done 18 months (+/−6 months) after cART initiation, which was the time point where most patients were represented.

The patients’ baseline data have been collected from medical records and the Danish HIV Cohort and are presented in table 1.

**Table 1:** Baseline data (18 months (+/−) cART initiation) of the 101 patients who entered the study in 1997–1998 separated into groups based on survival until 1/1-2010.

	Survivor group	Deceased group	
	Alive by 1/1-2010	Dead by 1/1-2010	
	(N = 84)	(N = 17)	p
Age at baseline (years)^a^	44 (36.25–51.75)	49 (43.25–51.75)	0.081
Gender (%male)	90.5 (N = 76)	100 (N = 17)	0.185
CD4 nadir (*10^9^/L)^a^	0.150 (0.046–0.250)	0.170 (0.044–0.240)	0.702
AIDS defining events (%)	26.2 (N = 22)	35.3 (N = 6)	0.444
Hepatitis C (%)	10.7 (N = 9)	17.6 (N = 3)	0.420
History of smoking (%)	57.1 (N = 48)	76.5 (N = 13)	0.137
Proviral DNA (/10^6^ PBMC)^a^	404.0 (207.0–774.5) (N = 77)	267.0 (150.0–1420.0) (N = 15)	0.996
CD4 count (*10^9^/L)^a^	0.340 (0.230–0.520) (N = 83)	0.325 (0.142–0.495) (N = 16)	0.492
CD8 count (*10^9^/L)^a^	0.880 (0.700–1.200) (N = 83)	0.950 (0.557–1.200) (N = 16)	0.438
Naïve CD4 cells (% of CD4 cells) a	29.4 (17.6–42.7) (N = 79)	19.5 (11.0–32.8) (N = 16)	**0.028**
Memory CD4 cells (% of CD4 cells) a	51.7 (40.2–64.9) (N = 78)	57.2 (45.5–64.8) (N = 14)	0.263
Activated CD4 cells (% of CD4 cells) a	18.2 (10.8–28.4) (N = 79)	16.1 (12.5–31.7) (N = 16)	0.783
Naïve CD8 cells (% of CD8 cells) a	21.8 (14.4–31.7) (N = 79)	19.5 (9.2–23.1) (N = 16)	**0.049**
Memory CD8 cells (% of CD8 cells) a	36.0 (23.7–44.0) (N = 78)	26.8 (21.7–32.5) (N = 14)	0.060
Activated CD8 cells (% of CD8 cells) a	17.2 (10.7–25.9) (N = 79)	15.6 (10.9–25.0) (N = 16)	0.950
Naïve CD4 cells (*10^9^/L) a	0.097 (0.038–0.17) (N = 79)	0.057 (0.018–0.14) (N = 16)	0.053
Memory CD4 cells (*10^9^/L) a	0.16 (0.11–0.22) (N = 77)	0.20 (0.091–0.26) (N = 14)	0.817
Activated CD4 cells (*10^9^/L) a	0.052 (0.028–0.10) (N = 79)	0.053 (0.036–0.074) (N = 16)	0.670
Naïve CD8 cells (*10^9^/L) a	0.15 (0.093–0.42) (N = 78)	0.085 (0.040–0.17) (N = 16)	**0.004**
Memory CD8 cells (*10^9^/L) a	0.28 (0.14–0.42) ( = 78)	0.19 (0.057–0.27) (N = 14)	0.121
Activated CD8 cells (*10^9^/L) a	0.12 (0.068–0.22) (N = 79)	0.091 (0.033–0.21) (N = 16)	0.442

a :median (interquartile range).

### T cell Analysis and Proviral DNA

The immunological parameters used in survival analysis (proportions of naïve, memory and activated CD4 and CD8 cells, and proviral DNA) are derived from blood samples collected 18 months after initiation of cART (+/−6 months).

T cell subsets were analyzed in fresh whole blood using 4-color flowcytometry and were defined as naïve CD4/8(CD45RA+, CD62+), memory CD4/8 (CD45RA-, CD45RO+) and activated CD4 (HLA-DR+), and activated CD8 (HLA-DR+, CD38+). Proviral HIV DNA copies per 10^6^ peripheral blood mononuclear cells were quantified by a prototype assay (Amplicor HIV DNA assay; Roche Diagnostic Systems), in accordance with the manufacturer’s recommendations.

All immunological and virological methods have been described in greater detail elsewhere [20], [21].

### Statistics

Statistical analyses were conducted using SPSS 11.5.

The 101 subjects were divided into two groups, a survivor group that consisted of patients who were alive by 1/1-2010 (n = 84) and a deceased group of patients who had died before this time (n = 17) (table 1).

For comparison of means in table 1, Chi Square or Fischer’s exact tests were used for categorical data as appropriate and independent variables T test was used for nominal data. Data were log 10 transformed to obtain normal distribution. Data are presented as medians and interquartile ranges.

In order to identify risk factors for overall mortality in the population Kaplan-Meier analysis was used to construct survival curves and Cox regression analyses to estimate hazard ratios (HRs). Person years at risk were computed in an observation period from the day of the patient’s blood sample 18 months after initiation of cART (+/−6 months) till 1/1-2010 or death, whichever came first.

**Figure 1:**
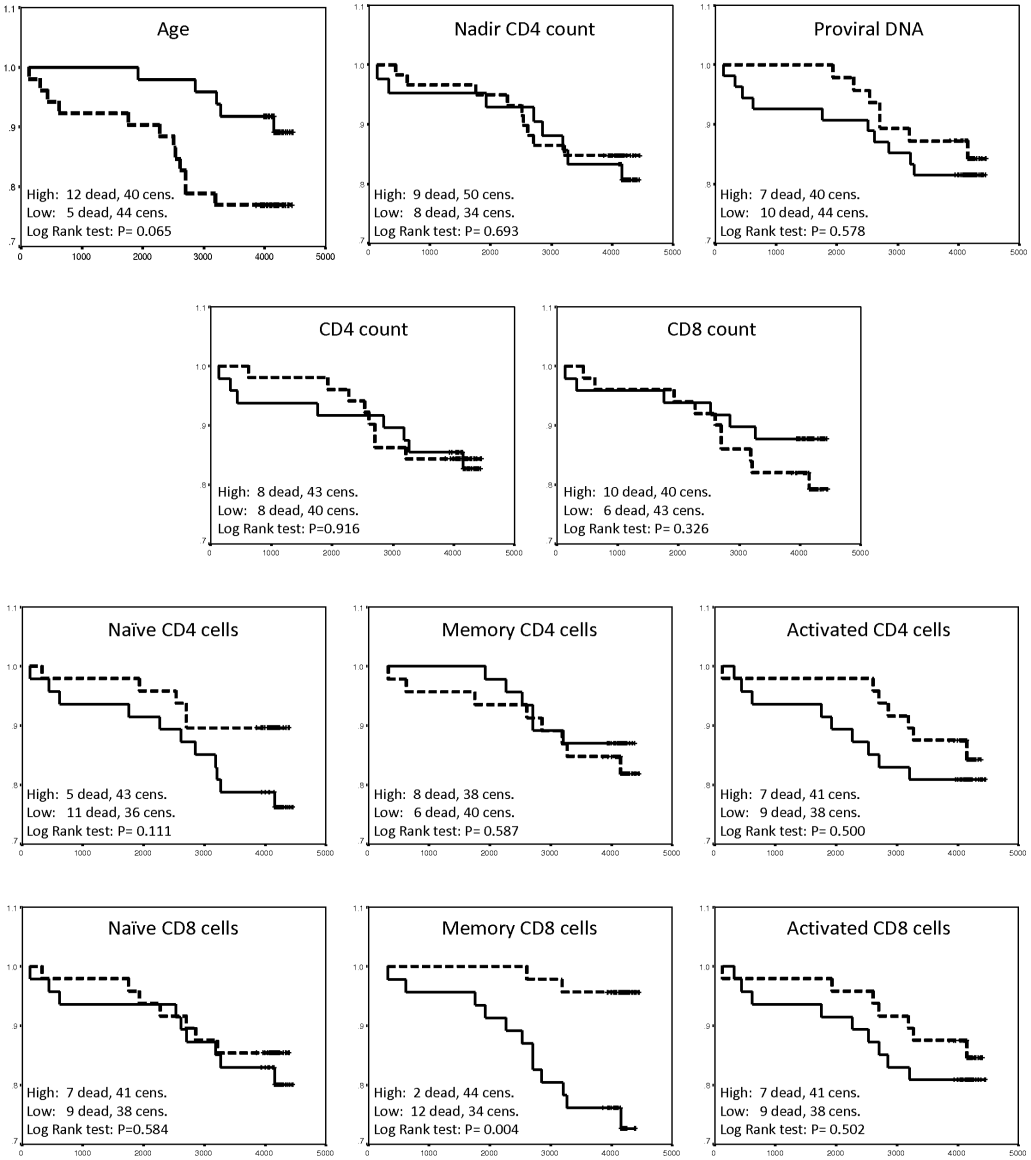
Kaplan-Meier plots. The observation period (days) is from 18 months (+/−6 months) after initiation of cART till death or 01/01/2010. The dotted line depicts the highest 50^th^ percentile and the full line depicts the lowest 50^th^ percentile. Analysis of survival linked to T cell subset are made with proportions of CD4+ and CD8+ cells respectively. Analysis using concentrations instead of proportions yielded similar results. Subsets were defined as naïve CD4/8(CD45RA+, CD62+), memory CD4/8 (CD45RA-, CD45R0+) and activated CD4 (HLA-DR+), and activated CD8 (HLA-DR+, CD38+).

Kaplan-Meier plots (Figure 1) were made by dividing the population into the highest and lowest 50^th^ percentile of the given variables. Analysis of survival linked to T cell subsets are made with proportions of CD4+ and CD8+ cells respectively.

Uni- and multivariate Cox regression analysis was preformed only for patients with complete data for multivariate analysis (n = 90). In the multivariate analysis the T cell subsets – given as proportions of CD4 or CD8 T cells respectively - were adjusted for age, CD4 count at the time of T cell subset measurement, nadir CD4 count, as well as AIDS and hepatitis C status (Table 2). Hazard ratios (HR), 95% confidence intervals (CI) and p-values are presented.

**Table 2:** Uni-and multivariate cox regression.

	Naïve %				Memory %				Activated %			
CD4		95% CI				95% CI				95% CI		
	HR	Lower	Upper	p	HR	Lower	Upper	p	HR	Lower	Upper	p
Univariate	0.9726	0.9407	1.0056	0.1026	1.0156	0.9845	1.0476	0.3296	0.9972	0.9644	1.0310	0.8675
Multivariate	0.9709	0.9369	1.0061	0.1039	1.0153	0.9791	1.0529	0.4117	0.9892	0.9490	1.0311	0.6078
Age	1.0698	0.9952	1.1500	0.0673	1.0730	0.9972	1.1546	0.0594	1.0761	1.0004	1.1575	**0.0487**
CD4	1.0030	0.9993	1.0066	0.1141	1.0024	0.9988	1.0060	0.1850	1.0020	0.9986	1.0054	0.2535
Nadir	0.9967	0.9904	1.0030	0.3031	0.9972	0.9909	1.0034	0.3735	0.9968	0.9902	1.0035	0.3502
AIDS	0.9153	0.2679	3.1268	0.8877	1.0362	0.2963	3.6240	0.9555	1.2340	0.3622	4.2044	0.7367
Hep C	3.7738	0.7316	19.4651	0.1126	3.9888	0.7635	20.8379	0.1010	4.4268	0.8491	23.0791	0.0774
	**Naïve %**				**Memory %**				**Activated %**			
**CD8**		**95% CI**				**95% CI**				**95% CI**		
	**HR**	**Lower**	**Upper**	**p**	**HR**	**Lower**	**Upper**	**p**	**HR**	**Lower**	**Upper**	**p**
Univariate	0.9707	0.9263	1.0173	0.2141	0.9676	0.9345	1.0019	0.0642	0.9941	0.9574	1.0321	0.7559
Multivariate	0.9657	0.9179	1.0159	0.1769	0.9509	0.9129	0.9906	**0.0157**	0.9914	0.9488	1.0358	0.6987
Age	1.0747	1.0001	1.1548	**0.0497**	1.0814	1.0051	1.1634	**0.0361**	1.0719	0.9967	1.1528	0.0613
CD4	1.0024	0.9988	1.0060	0.1866	1.0020	0.9989	1.0052	0.2092	1.0019	0.9986	1.0052	0.2639
Nadir	0.9966	0.9902	1.0031	0.3079	0.9949	0.9880	1.0018	0.1442	0.9971	0.9909	1.0035	0.3758
AIDS	0.9713	0.2795	3.3752	0.9634	1.5467	0.4250	5.6285	0.5082	1.2771	0.3710	4.3954	0.6982
Hep C	4.0645	0.8260	20.0005	0.0846	4.2375	0.8398	21.3813	0.0804	4.3287	0.8312	22.5421	0.0818

The analysis only included subjects with complete data (N = 90).

HR: hazard ratio, CI: confidence interval, %: proportion of total CD4 or CD8 cells respectively.

Subsets were defined as naïve CD4/8(CD45RA+, CD62+), memory CD4/8 (CD45RA-, CD45R0+), activated CD4 (HLA-DR+), and activated CD8 (HLA-DR+, CD38+).

Nadir CD4 count was defined as the lowest CD4 cell count measured before or during the first 18 months of cART.

P-values <0.05 were considered significant.

## Results

### Baseline Characteristics

One-hundred-and-one patients entered the study in 1997-98. On the 1/1-2010 seventeen of those had died.

The baseline data of the two groups (Table 1) differed somewhat in that the deceased group tended towards having more independent risk factors of morbidity and mortality; higher age, more smokers, higher prevalence of AIDS and hepatitis. Eighteen months after cART initiation the two groups showed largely similar total CD4 and CD8 counts, but the deceased group had a significantly lower proportion of CD4 and CD8 cells being naïve, and a lower concentration of naïve CD8 cells. Significant differences were not observed with respect to CD8 memory cells or activated CD8 cells but the deceased group tended to have lower concentrations of these subsets.

All patients had HIV RNA measured approximately 4 times a year as part of standard care and in the survivor group measurements were below detectable limits in a mean of 81% of measurements vs. 72% in the deceased group.

### Mortality

The causes of the seventeen deaths were: non-AIDS cancer (n = 5), cardiovascular disease (n = 5), hepatitis C (n = 3), one case of suicide, one case of pneumonia, one case most likely linked to alcoholism, and only one AIDS related case which was non-Hodgkin lymphoma. Three of the deceased patients had hepatitis C and they all died from liver failure due to this disease.

In the survivor group there were two patients with a history of malignant non-AIDS cancer and two patients with a history of acute myocardial infarction.

### T Cell Subsets and Relation to Survival

Kaplan-Meier survival analysis (Figure 1) revealed significantly lower mortality among the patients who belonged to the highest 50^th^ percentile of memory CD8 cell (CD45RA-,CD45RO+) proportions (P = 0.004). Nadir CD4 count, proviral DNA, and total CD4 and CD8 counts did not influence on mortality.

Multivariate Cox regression (Table 2) confirmed the protective effects of high proportions of memory CD8 cells, with a HR of 0.95 (P = 0.016), reflecting that a one percentage-point increase in CD8+CD45RO+CD45RA- T cells decreased the relative risk of death during follow up by 5%. The same CD8 subset was borderline significant in the univariate model (HR = 0.97, P = 0.064).

When removing the three deceased patients who died of suicide, alcoholism and AIDS/lymphoma respectively these results remained largely unchanged (Kaplan Meier log rank test: p = 0.0032, multivariate Cox regression: HR = 0.944, p = 0.019).

Age was a significant factor in several of the multivariate models with HR = 1.07−1.08, but none of the chosen adjusting factors were univariately significant.

## Discussion

The present study exclusively included patients who had immunological studies done during fully suppressive cART and followed them for more than 10 years. The main findings were i) a high level of memory CD8+ T cells, defined as CD45RA-CD45RO+, was an independent predictor of increased overall survival and ii) immune activation defined as proportions of CD4+HLA-DR+ and CD8+HLA-DR+CD38+ showed no prognostic value.

The causes of death among the subjects in the present study are largely consistent with those found in a Danish nationwide study of patients who were virally suppressed for more than 3 years, in which the primary causes of death were cancer, sudden death of unspecified cause, and liver disease, and very few deaths were due to AIDS related illness [22]. A large multicenter, multinational study reported 435 deaths in 11,593 HIV-infected and found similarly only 10% of death related to AIDS defining disease, primarily non-Hodgkin lymphoma, and 21% related to non-AIDS cancers, 9% cardiovascular disease, and 9% liver disease [23].

It is generally accepted that CD8 T cells play a role in controlling untreated HIV infection, but the exact role of CD8 cells displaying memory phenotype (CD45RA-CD45RO+) is only incompletely understood during cART.

As the CD8+CD45RA-CD45RO+ subset in this study is not sub-characterized further, the specific subtype and function cannot be determined, and the subset most likely represents a mixture of early, antigen experienced CD8 cells, including central memory, effector memory and HIV specific CD8 cells (CD45RA+/−CD28-CD27+CCR7-) with specific cytokine releasing properties [24], [25]. Therefore it is not possible to conclude from these data which subtype of memory CD8 T cells is associated with reduced mortality.

In accordance with the present results we have previously reported a protective effect of high CD8+CD45RA-CD45RO+ levels among untreated patients [26]. However a recent study found no prognostic value of central memory CD8 T cells defined as CD45RO+CD28+CCR7+CCR5- in treatment naïve patients, but a decreased risk of disease progression and death with higher levels of CD127+ CD8 T cells [27] that are important for homeostasis of T cells and differentiation into memory CD8 phenotype [28], [29].

Our results failed to demonstrate the expected positive association between early immune activation and all-cause mortality. High levels of CD4 and CD8 cells expressing the activation markers CD38 and HLA-DR did not show any prognostic value.

Most previous studies that have shown prognostic value of cellular immune activation markers differ from this study in that they primarily included treatment naïve patients as well as treated patients, were cross sectional or with shorter follow up periods.

A study from Uganda recently showed that higher proportions of CD8+CD38+HLA-DR+ T cells six months after initiation of successful treatment was an independent predictor of increased mortality, but the same subset pre-cART had no significant impact [30]. However, the samples were taken earlier after cART at a time when pre-cART immune activation could still be declining, the follow up was shorter, and the mortality significantly higher suggesting differences in causes of death and the frequency of co-infections i.e. tuberculosis.

It has been suggested that even in the well controlled cART treated HIV infection the immune system has similarities to that of old people and it has further been suggested that these changes formed the basis for the morbidity and mortality observed [31]. These changes include poor T-cell regeneration, elevated levels of activated CD38+HLA-DR+ T cells and accumulation terminally differentiated CD8 memory cells [32]–[34]. We did find that those who died during follow-up had fewer naïve cells - significantly different for CD4 cell and borderline for CD8 cells (Table 1) – but these findings were not significant in survival analysis, and neither immune activation nor expansion of CD8 cell subsets were associated with increased risk of death.

The strengths of this study are the un-interrupted, long-term treatment of the patients, a very long observation period combined with the accessibility of data on T cell subsets during cART. The weaknesses are the relatively small number of patients and the broad definitions of T cell subsets, primarily due to the limited amount of colors available for flowcytometry at the time, and the absence of data on the functional properties of the cells. Due to these factors the presented data should be interpreted with caution.

In summary, the study demonstrated a significant survival benefit of high levels of CD8+CD45RO+CD45RA- T cells. T cell activation as defined as CD4+HLA-DR+ and activated CD8+ CD38+HLA-DR+ did not show prognostic value in this group of long term viraemically suppressed HIV patients.

Larger and longitudinal studies including more specific T-cell subset analyses such as CD28 and CD57 are needed to accurately establish prognostic factors, but overall the results suggest that prognostic information exists within the CD8 compartment.
